# Excess of Mortality in Adults and Elderly and Circulation of Subtypes of Influenza Virus in Southern Brazil

**DOI:** 10.3389/fimmu.2017.01903

**Published:** 2018-01-08

**Authors:** André Ricardo Ribas Freitas, Maria Rita Donalisio

**Affiliations:** ^1^Department of Social Medicine, School of Medicine San Leopoldo Mandic, Campinas, Brazil; ^2^Department of Public Health, School of Medical Sciences, University of Campinas, Campinas, Brazil

**Keywords:** influenza, excess of mortality, influenza AH3N2, influenza AH1N1, pandemic, elderly, Serfling regression model

## Abstract

**Purpose:**

In the elderly population, the influenza infection and its clinical complications are important causes of hospitalization and death, particularly, in longer-lived age. The objective of this study is to analyze the impact of influenza virus circulation on mortality in the elderly and adults, in years with different predominant virus strains.

**Methods:**

We performed a time trend study to evaluated excess of mortality for pneumonia and influenza, respiratory disease, and all-causes in southern region of Brazil, from 2002 to 2015. After considering other models, we opted for Serfling regression. Excess of death rates per 100,000 inhabitants were analyzed in specific age groups (24–59, 60–69, 70–79, ≥80 years) and by year of occurrence. Mortality information were taken from Brazilian Mortality Information System and etiological data were accessed in Sentinel Virological Surveillance database, getting the weekly positivity of the immunofluorescence tests for influenza A (H1N1, H3N2), and B.

**Results:**

In southern Brazil, there is an evident seasonal pattern of all death outcomes among different age groups in the dry and cold season (April–September). The highest excess mortality rates occurs among older, particularly in years of circulation of influenza AH3N2, especially among people ≥80 years, in 2003 and 2007—years of great severity of influenza activity. After 2009, with the introduction of the pandemic influenza AH1N1, we observed a lower impact on the mortality of the elderly compared to <60 years.

**Discussion:**

A cross reactivity antibody response from past exposure probably provided protection against disease in the elderly. Despite not controlling for comorbidities, climate, and vaccination, for the >70 years, ratio of respiratory diseases excess mortality rates between AH1N1 (2009) and severe year of H3N2 (2007) shows protection in the pandemic year and great vulnerability during AH3N2 virus predominance.

**Conclusion:**

The reduced immune response to infection, and to vaccination, and presence of comorbidities recommend a special attention to this age group in Brazil. Besides medical assistance, the timeliness of vaccine campaigns, its composition, and etiological surveillance of respiratory diseases are some of the preventive and public health measures.

## Introduction

Human influenza viruses can cause diseases through many direct and indirect pathological effects. Consequences are destruction of infected cells, release of cytokines leading to fever, malaise, damage to respiratory epithelium and pulmonary parenchyma, and pneumonia. It includes secondary bacterial infections because of tissue damage and exacerbation of preexisting comorbidities such as cardiovascular and renal diseases, diabetes, or chronic lung disease (1–3).

The rates of hospitalization and mortality associated with influenza are higher among patients with chronic diseases, children under 1 year and after 65 years of age (4, 5). With the aging population in recent decades, the raw number of hospitalizations and deaths related to pneumonia and influenza tends to increase (4), this phenomenon has been observed also in Brazil (6, 7). However, the impact and severity of influenza virus circulation depend in part, on the strain that predominates in the season each year.

Due to the lack of laboratory confirmation, influenza-associated morbidity and mortality are often classified as pneumonia, other respiratory diseases, or other causes. Given the difficulty of directly measuring influenza morbidity and mortality, time series models are used to elucidate disease patterns in various age groups. Trends are usually determined by means of statistical inference, based on seasonal coincidence of the occurrence of certain diseases or death and laboratory confirmation of the viral circulation (4, 8).

Different approaches, with and without the quantification of the proportion of viral isolates, can produce average estimates of excess deaths associated with the circulation of certain viral variants (9–11). Viral surveillance data, hospitalization, or death indicators are particularly useful for the study of influenza in the tropics, as seasonality may be less evident (11–13). Serfling regression has been used to analyze excess of mortality related with respiratory virus circulation (7, 14–16). Despite some limitations (17), the inclusion of sinusoidal terms in weekly regression may reduce spurious correlation between influenza occurrence and death (18, 19). It is particularly useful when no other covariables are available, and with small samples of viral sentinel surveillance data (18). Poisson regression and the generalized linear model (GLM) can produce more specific estimates and support adjustments for variables (temperature, humidity, comorbidities, other circulations of viruses), although they require a more robust and consistent virological surveillance and cannot be used for pandemics (4).

In Brazil, surveillance for influenza syndromes was implemented in 2000, monitoring the occurrence of respiratory viruses (influenza A and B, parainfluenza 1, 2, and 3, respiratory syncytial virus, adenovirus). The Brazilian Ministry of Health provides vaccination coverage annually since 1999 for seniors and some risk groups, with vaccine coverage of the elderly population at around 80% in southern Brazil, the region with the highest coverage of the country. Despite the adequate coverage, protective titers after vaccination (HI ≥ 0) are consistently lower with poorer cell mediate and antibody responses in the elderly comparing to adults (20).

Considering the vulnerability of the elderly to influenza virus infection, and the lack of studies on its repercussion in Brazil, the objective of this study was to analyze the impact of different strains of Influenza A virus circulation. We analyzed particularly the most predominant variants (AH1N1 and AH3N2) on excess of mortality in the adults and elderly of different age groups in a region with marked seasonality of respiratory diseases in Brazil.

## Materials and Methods

### Local of Study

This is a time trend study to evaluated excess of mortality from 2002 to 2015 in southern region of Brazil (states of Paraná, Santa Catarina e Rio Grande do Sul), total area is 576,774,31 km^2^, population is 27,386,891 inhabitants with subtropical climate (Köppen-Geiger classification Cfa). We choose these states for analysis because of the consistent seasonal pattern of influenza, as well as the availability and quality of etiological data from the virological surveillance system in that region.

### Mortality Data and Population

For the mortality rates of specific age groups (24–59, 60–69, 70–79, and ≥80 years) and death causes, we took data from Brazilian Mortality Information System. Causes are classified according to International Causes of Death ICD-10 revision, pneumonia, and influenza (ICD J 10 to J18.9), respiratory diseases (ICD J00 to J99), and all-cause (excluding external causes of mortality).

We obtained population of each year and age group from Instituto Brasileiro de Geografia e Estatística-IBGE from the Census-2010, and population estimates for the following years.

### National Viral Surveillance Data

Etiologic information of flu-like syndrome was accessed in database of the National Sentinel Virological Surveillance System. It has data from 128 sentinel units distributed in all regions of the country—North (21 units), Northeast (26 units), Southeast (34 units), South (38 units), and Central West (9 units). Surveillance is performed through the systematic collection of weekly samples of nasopharyngeal secretions from patients who present flu-like syndrome. Reference laboratories process samples by using indirect immunofluorescence (IIF), with tests for influenza A and B, parainfluenza 1, 2, and 3, respiratory syncytial virus, and adenovirus. A portion of the samples is submitted to polymerase chain reaction tests to identify the virus genotype.

We calculated the laboratory positivity indicator using weekly positive results of IIF divided by the total of weekly valid tests, i.e., excluding the results within inadequate samples (not enough biological material, improper storage, incorrect material in the sample) or inconclusive results (no valid results).

Influenza vaccination coverage (%) of southern region from 2002 to 2015 was obtained from Brazilian National Program of Immunization data base (DATASUS).

### Definition of Influenza Epidemic Periods

The criteria used to define the period of increase of influenza activity was when the positivity of the samples tested exceeded twice the annual mean of the weekly positivity of samples processed by surveillance, during two consecutive weeks.

In the year 2009, we consider the period officially recognized by the Brazilian Ministry of Health as epidemic by the influenza AH1N1pmd2009 strain, due to irregularity of the sample collection by the sentinel surveillance system at the end of epidemic.

### Statistical Analysis

We calculated the weekly mortality rates by age group using the number of deaths per group of causes divided by the estimated population in the middle of the year multiplied by 100,000.

We constructed a Serfling cyclical regression model (14) for weekly data applied to each age group and causes of death (pneumonia and influenza, respiratory diseases, and all causes), as seen in others studies (7, 15), to estimate baseline of predicted deaths in the absence of influenza epidemics.

To fit regression, we used period of 13 years (from 2002 to 2015), excluding the weeks of epidemics periods. A cyclical linear regression was adjusted with the equation:

$$\begin{array}{c} Y=\text{β}0+\text{β}1*t+\text{β}2*t2+\text{β}3*t3+\text{β}4*\text{sin}(2*\pi *t/52.17)\\ \;+\text{β}5*\text{cos}(2*\pi *t/52.17)+\text{β}6*\text{sin}(4*\pi *t/52.17)\\ \;+\text{β}7*\text{cos}(4*\pi *t/52.17)+e1, \end{array}$$

where *Y* is the mortality rate, β is the coefficients of regression, *t* is time in weeks, and *t*^2^ and *t*^3^ are variables for adjusting the secular trend of the disease. We used of sine and cosine for adjust of annual and semiannual periodic components.

After adjusting a linear regression and define the expected mortality rate, we delimited 95% upper confidence limit of the baseline as the reference threshold in the absence of influenza epidemics. We calculated the excess of deaths as the observed mortality minus the expected mortality in the periods when mortality was above 95% of the confidence interval during epidemics periods.

We also present ratios of excess mortality rates among years of predominant circulation of influenza strains AH3N2 (mean and years of severity), AH1N1 pre-pandemic, and AH1N1 post-pandemic for each age group.

For data compilation, we used Microsoft Office Excel 2007, and for statistical analysis, SPSS for Windows, version 24.0.

## Results

Table 1 shows the proportion of positivity of the IIF nasopharyngeal samples and the annual prevalence of strains of influenza in the period. Before 2009, the year of entry of the pandemic strain AH1N1pmd 2009, there was a predominance of influenza AH3N2 in the years 2003 to 2007. After 2009, there is alternation of strains in the southern Brazil. Annual elderly vaccination coverage in southern region is high and homogeneous, around 80%, and even higher in the recent years.

**Table 1:** Specimens collected, positive proportion, and predominant subtypes of influenza between 2002 and 2016 in the sentinel units of southern Brazil.

Years	Samples	Positive samples	% indirect immunofluorescence positive	AH1N1	AH3N2	Not subtyped	B	Virus Predom.	% Vac. Coverage
2002	186	23	12			8	15	H1/B	75.3
2003	355	41	12			37	4	H3	73.0
2004	474	52	11			47	5	H3	75.6
2005	383	15	4			14	1	H3	72.7
2006	676	94	14			72	22	H3	84.0
2007	800	127	16			122	5	H3	71.1
2008	1,018	129	13			60	69	H1/B	71.4
2009	540	112	21			91	21	H1/B	76.9
2010	949	142	15			62	80	H1/B	77.1
2011	1,104	175	16			129	46	H3	82.9
2012	1,514	138	9			106	32	H1/B	96.5
2013	3,728	926	25	320	229	0	377	H1/B	91.8
2014	5,249	815	16	73	660	10	72	H3	91.4
2015	5,752	854	15	109	390	19	336	H3	93.7

**Figure 1:**
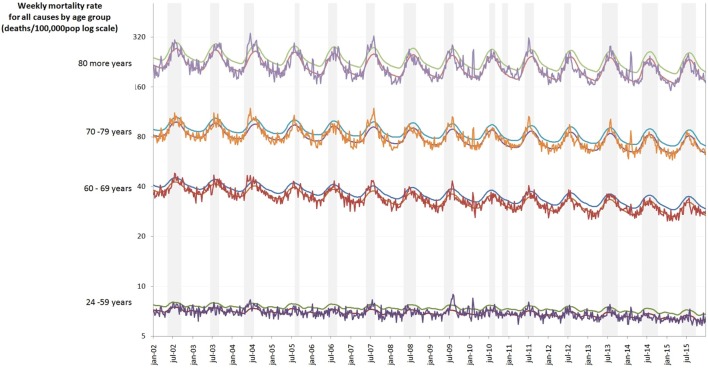
Weekly mortality rate by age group (deaths/100,000) log scale in southern regions of Brazil, 2002–2015. Colored lines are age groups and gray columns are the seasons of influenza.

There is an evident seasonal pattern of deaths from pneumonia and influenza, respiratory diseases, and all-causes among the elderly in different age groups in the dry, cold months (April–September) in southern region (Figure 1).

We note a progressive increase in the rates of excess deaths (of all outcomes) with increasing age, especially among those older than 70 years. In the pre-pandemic years with dominance of the AH1N1 strain, the excess of mortality rates associated with influenza were relatively low, compared to years of prevalence of AH3N2 strain (Table 2). Among those over 80 years, the ratio of excess mortality rates between 2009 and the years with dominium of H3 strains was less than one. This ratio suggests that this age group was spared in the 2009 pandemic. However, in years of predominance of strain H3, excess of mortality rate of all causes in this group were 449.6 per 100,000 (corresponding to 1,598 obits), 5, and 8.2 times greater than the same rate in years of circulation of H1N1 in pre- and post-pandemic period, respectively.

**Table 2:** Excess mortality rate (per 100,000 population) and excess deaths (absolute number) according to influenza virus subtypes prevalent in southern Brazil, 2002–2015.

		24–59		60–69		70–79		≥80	
		Excess mort rate	Exc. deaths	Exc. mort rate	Exc. deaths	Exc. mort rate	Exc. deaths	Exc. mort rate	Exc. deaths
Mean of annual excess mortality during all period	Pnm and Flu	0.2	27	0.8	17	2.9	29	20.2	85
	Resp	0.4	57	3.8	64	12.3	113	37.2	143
	All causes	1.3	176	13.0	214	37.0	331	116.5	422
Mean of annual excess mortality in H1/B pre-pandemic years (2002 and 2008)	Pnm and Flu	0.0	0	0.4	7	2.1	19	2.2	9
	Resp	0.0	0	3.5	54	7.4	59	15.4	42
	All causes	0.9	118	13.1	201	35.7	287	88.3	255
Excess mortality during 2009	Pnm and Flu	2.5	331	3.2	57	12.0	113	20.5	82
	Resp	5.2	698	7.4	129	17.6	167	7.3	29
	All causes	7.1	953	21.0	368	58.6	555	125.1	501
Mean of annual excess mortality in H1/B pos-pandemic years (2010, 2012, and 2013)	Pnm and Flu	0.4	60	1.5	31	4.2	44	17.3	83
	Resp	0.7	99	3.0	62	14.3	150	21.5	102
	All causes	0.9	129	5.6	116	16.6	178	54.6	262
Mean of excess mortality during H3 years (2003, 2004, 2005, 2006, 2007, 2011, 2014, 2015)	Pnm and Flu	0.0	4	0.6	12	2.1	22	27.4	112
	Resp	0.1	13	4.1	66	12.6	111	50.6	191
	All causes	1.2	148	15.1	240	42.3	371	145.6	514
Excess mortality during severe H3 epidemics (2007)	Pnm and Flu	0.0	0	0.6	10	2.1	19	49.5	176
	Resp	0.2	24	8.5	138	32.5	288	134.2	477
	All causes	2.9	375	35.6	576	136.1	1,209	449.6	1,598
Relative risk 2009/mean of period	Pnm and Flu	12.6		3.8		4.1		1.0	
	Resp	12.4		1.9		1.4		0.2	
	All causes	5.3		1.6		1.6		1.1	
Relative risk 2009/mean of H3 years	Pnm and Flu	83.9		5.6		5.6		0.7	
	Resp	49.2		1.8		1.4		0.1	
	All causes	6.2		1.4		1.4		0.9	
Relative risk 2009/severe H3 epidemics	Pnm and Flu	#		5.5		5.6		0.4	
	Resp	28.3		0.9		0.5		0.1	
	All causes	2.5		0.6		0.4		0.3	

*Exc., excess; Pnm and FLU, pneumonia and influenza; Resp, Respiratory causes International Causes of Deaths ICD chapter J (J10–J18.9)*.

*# - Relative risk has no valid value as denominator is zero*.

Among adults (24–59 years), we observe a large excess of deaths rates during the 2009 pandemic (953 obits), which correspond to 7.1 excess deaths from all causes, and 99 excess mortality from respiratory diseases associated with viral infection in every 100,000 individuals of the age group. The ratio between excess mortality rates due to pneumonia/influenza in the pandemic year (2009) and the mean rate of the period was 12 times higher among the youngest (Table 2).

Rates of excess mortality by pneumonia and influenza and respiratory diseases are lower than all causes in all age groups, but particularly high in older than 80 years (Table 2).

## Discussion

The results highlight the great vulnerability of elderly to influenza AH3N2, especially among older than 70 years in severe years of influenza activity, like 2003 and 2007. The study also shows the lower impact of influenza AH1N1pdm 2009 in this age group compared to younger. Risk of dying among the elderly in years of circulating AH3N2 influenza has been reported in several parts of the world (9, 10, 21, 22); however, in Brazil, there are no recent estimates available. Few studies analyze the circulation and impact of influenza in tropical and subtropical regions (6, 7, 9–11). Influenza B virus is also associated with severe disease (23); however, this variant did not circulate with intensity during the study years in Brazil.

Although the elderly are the most vulnerable group to viral respiratory infections, we found relative small excess of deaths in years of circulating AH1N1 pre pandemic (2002 and 2008). Study comparing excess deaths from respiratory diseases in the elderly in Latin America shows stable rates (mean of 89.4 per 100,000 inhabitants) in southern Brazil between 1998 and 2008 (pre-pandemic Flu A-H1N1), although higher in Brazil than in other countries (24). In the USA and in European countries, influenza seasons dominated by subtype AH3N2 are typically associated with mortality two to three times higher than in seasons with predominance of AH1N1 (prior to pandemic strain 2009) and of influenza B viruses (9, 10, 19, 25).

When all causes of death are studied, the overall mortality associated with influenza among elderly exceeds that observed in younger age group. It should be considered that all causes mortality is a non-specific measure and a distant outcome of influenza infection. However, it is difficult to determine which group of causes of death could better characterize the influenza burden in mortality. By choosing only the respiratory causes, we may underestimate clinical complications of pulmonary viral infection (e.g., cardiovascular). Therefore, in this study, we analyzed all causes, respiratory, and pneumonia and influenza deaths.

The unfavorable evolution of infection in the elderly is possibly due to the prevalence of comorbidities, deficiencies in defense mechanisms, and poor antibody response to vaccination, as cell-mediated and humoral responses limit severity of disease (26). Patients with chronic diseases are more susceptible to infection due to decline of the immune function through inflammatory mechanisms, hindering the mucosal barrier, and the adaptive and innate immunological defense mechanisms (27).

The immune response to infection in the elderly tend to be delayed and weak, with prolonged inflammatory responses, which involves different types of host reaction, mainly to clearance virus. The exacerbations of these mechanisms may induce immune-mediated pathology causing tissue damage (28). Cytokine high serum levels of IL-6, TNF-a, IFN-g and sIL-2R, chemokines IP-10, MCP-1, and monokine induced by IFN-g (MIG), are associated with severe clinical cases and lung damage (29).

Immunological abnormalities in people with diabetes, chronic respiratory diseases, cardiopathy, or other chronic diseases have increased risk of severe infection and bad prognosis (19). For example, there is the consistent association of influenza infection with cardiovascular mortality, particularly acute myocardial infarction (30). In part, it is attributed to altering endothelial function due to an acute inflammatory and procoagulant stimulus during viral infection (31, 32). Clinical complications of diabetes triggered by influenza infection cause impairment of leukocyte function and increase post-infection colonization rates resulting in poor prognosis in the elderly (33, 34).

In young people and adults, in 2009, the emerging influenza AH1N1 strain had a notable impact on the mortality of people up to 59 years in various parts of the world, including Brazil (7, 25, 35, 36). Excess mortality of individuals aged 24–59 years in the state of São Paulo, Brazil was identified during the pandemic AH1N1 virus (7). Pregnant women adults with metabolic conditions, including obesity, chronic respiratory disease, and other chronic diseases were significantly associated with severe acute respiratory syndrome and the lethality in Brazil (37). Our study showed a 41.5-fold higher rate of mortality from pneumonia and influenza in adults (24–59 years) in the pandemic year AH1N1 than the average of years with predominance of AH3N2 circulation in southern region.

In addition to the clinical severity and the large portion of the affected population, pandemics affect age groups in different ways (38). While only 10% of deaths from seasonal influenza occur among those under 65 years of age, in the pandemics of 1918, 1957–1958, and 1968, this proportion was 95, 40, and 50%, respectively (39). Therefore, pandemics tend to affect a larger proportion of young people than seasonal influenza. In this study, higher rates of death due to pneumonia, influenza, respiratory, and all causes were observed among those aged 24–59 years in 2009.

One explanation for the higher mortality observed among the youngest is that they would be more prone to the situation known as “cytokine storm,” i.e., a dysfunctional overproduction of cytokines that would lead to diffuse damage to the respiratory tract with severe and potentially lethal systemic repercussions (40). Viral replication and production of inflammatory mediators seem to be involved in the pathogenesis of infection with influenza A H1N1pmd2009, hindering the clearance of virus in lung tissue and leading to pathologic lesions (41).

Another explanation for the lower mortality in the elderly is that they were exposed previously to antigens of the pandemic virus. Hancock et al. (42) suggested a cross-reactive antibody response to 2009 pandemic AH1N1. Similarities between AH1N1 antigen from 2009 and 1918 were detected. This last virus strain has not circulated since 1958 (39), when the AH1N1 strain was displaced by AH2N2 (Asian flu). At that time, AH1N1viral circulation occurred mainly in children, the current elderly of 2009.

The emergence of the AH3N2 strain in the pandemic year 1968 (Hong Kong flu) affected several age groups. This new strain resulted from a large genetic mutation (shift) recombining virus material of the circulating AH2N2 with the avian H3, of Asian origin, resulting in the new variant AH3N2 (38).

In 2002–2003, under selective pressure an antigenic small mutation (drift), resulted in A/Fujian/411/02(H3N2) a strains emerged after a “jump” in genes evolution of Hemagglutinin and Neuraminidase proteins of virus surface (43, 44). The circulation of the Fujian strain had a great impact on the mortality from pneumonia in several parts of the world in 2003–2004 and 2004–2005 (22) and in Brazil (45). In 2007, a new drift resulted in influenza AH3N2 detected in south Brazil (46) also affecting hospitalizations and deaths in various parts of the world (47). We observed high rates of excess mortality in the elderly, in the years of 2003 and 2007.

Limitations of this study refer mainly to the ecological analysis of pooled data. We did not analyze individual information regarding comorbidities and history of vaccination that could be important confounders influencing mortality (17). We just had the overall annual vaccination coverage which were in general, around 80% in the period. Estimates of the number of deaths (all causes, respiratory, and pneumonia-influenza) supposedly related to influenza may be inaccurate in inferring the impact of respiratory viruses. Correlations in time series studies may produce spurious associations, especially between all causes of death and influenza infection, due to the distance between cause and outcome, and to multiple components of the obits. Serfling addresses part of this limitation by introducing sinusoidal terms in equation, since non-influenza mortality is not expected to coincide exactly with sinusoidal pattern (14, 19). Moreover, excess mortality of pneumonia, respiratory diseases, and all causes can be considered as an alert to surveillance of viral respiratory diseases, such as a sentinel indicator to be investigated (4, 48). Although all causes mortality is a non-specific indicator, it does not underestimate the complications of chronic diseases associated with influenza (4). Despite the influenza component in all causes mortality is small, the indicator can be considered an indirect measure, a warning, useful in epidemiological monitoring.

Another limitation is the lack of robust etiologic data from virological surveillance in the years 2002–2012, which could lead to imprecision in the analyses; however, the data on the predominance strains in the southern region are reliable, and influenced the composition of the vaccine of each season.

Considering the option for the analysis model, Serfling linear regression may produce different estimates when compared with other models (Poisson, ARIMA, and GLM) (9, 10); Poisson and ARIMA models produce higher mortality estimates than Serfling, and Serfling higher than GLM, especially among the elderly (16, 17, 21). We chose Serfling model because we do not have robust virological surveillance data, before 2013, and the study period includes a pandemic year (4).

Besides, in this study, we did not analyze climatic variables (minimum temperatures and relative air humidity) that could also interfere with viral transmission and increase the impact of the disease, particularly in the elderly.

In conclusion, probably previous exposures to influenza AH1N1 in the past influenced the mortality of Brazilian elderly in 2009, despite the vulnerability of this age group to clinical complications. For the >70 years, we observe higher excess mortality rates (of all outcomes) in severe year of AH3N2 circulation (2003, 2007). It is also worth noting that vaccination has been associated with the prevention of death particularly at age 65 (49). Therefore, the high elderly vaccination cover in southern Brazil may have attenuated excess of mortality estimated, although the immune response is limited among those.

More attention should be given to the circulation of influenza AH3N2 in subtropical regions in Brazil. The reduced immune response to infection and to vaccination, and associated comorbidities recommend a special attention to this age group. Besides medical assistance, the timeliness of vaccine campaigns, its composition, and etiological surveillance of respiratory diseases in the region are some of the preventive and public health measures.

## Author Contributions

Both authors made contributions to the conception of the work, acquisition, analysis, interpretation of data, and writing the manuscript.

## Conflict of Interest Statement

Authors declare that the research was conducted in the absence of any commercial or financial relationships that could be construed as a potential conflict of interest.
